# Supplementary figures and images for: PDE5 inhibitor potentially improves polyuria and bladder storage and voiding dysfunctions in type 2 diabetic rats
Source: PLoS One. 2024 Sep 18;19(9):e0301883. doi: 10.1371/journal.pone.0301883 (PMC11410213; doi:10.1371/journal.pone.0301883)

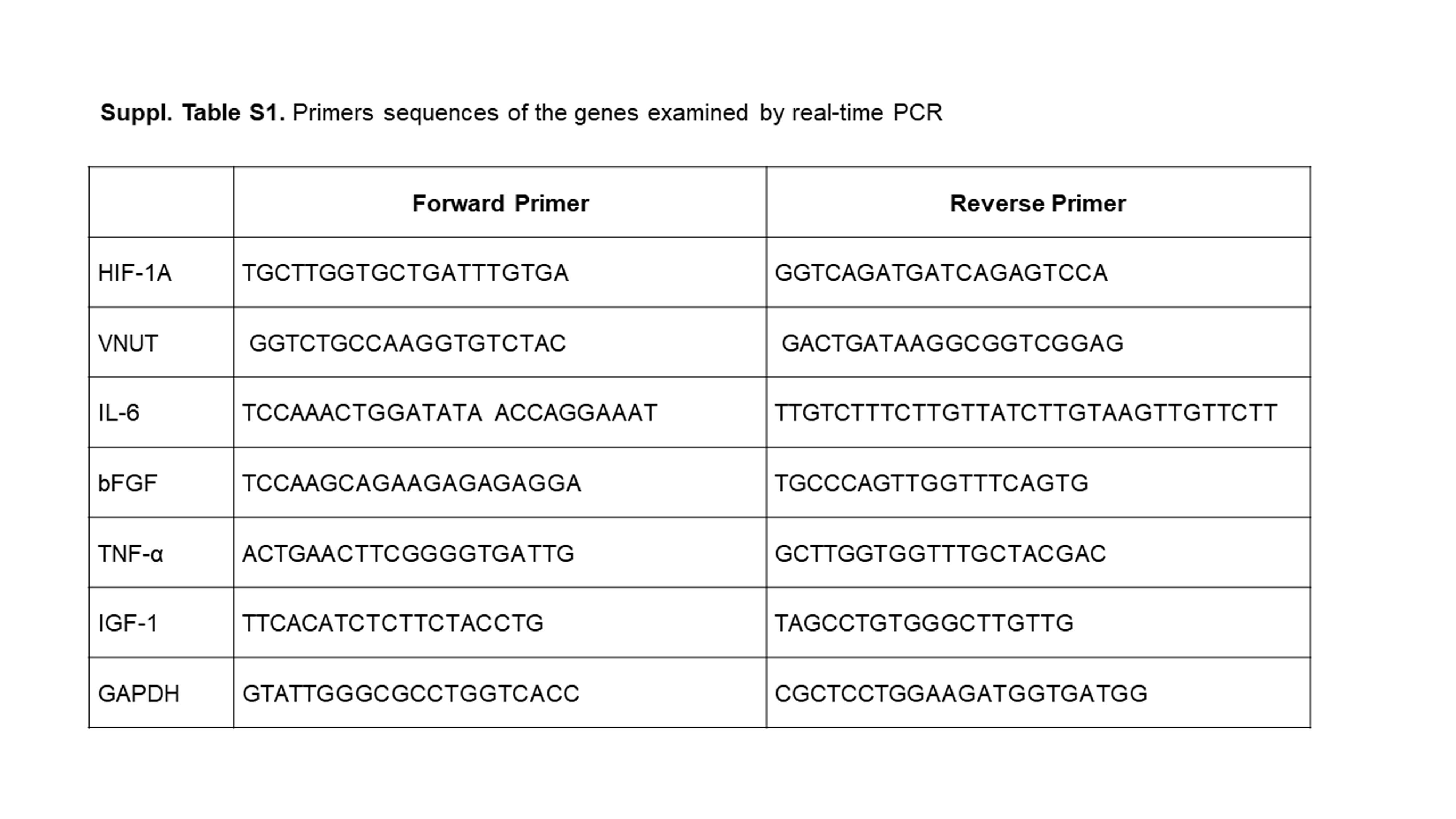

Supplement: S1 Table — (TIF) [file pone.0301883.s001.tif]
