# Supplementary material for: PDE5 inhibitor potentially improves polyuria and bladder storage and voiding dysfunctions in type 2 diabetic rats
Source: PLoS One. 2024 Sep 18;19(9):e0301883. doi: 10.1371/journal.pone.0301883 (PMC11410213; doi:10.1371/journal.pone.0301883)
Supplement: Attachment — Submitted filename: PONE-D-24-11736R1.docx [file pone.0301883.s002.docx]

The authors have now provided key research data, namely the total urine volume and bladder capacity.

These data are very helpful to explore mechanisms underlying the voiding phenotype of T2DM model (OLETF) rats, but raised a serious concern about the authors’ interpretation/conclusion. Thus, increased voiding frequency could be simply due to polyuria but not bladder dysfunction.

OLETF rats had x2.2 total urine volume and x1.8 voiding frequency at 36 weeks, x4.4 total urine volume and x3.9 voiding frequency at 48 weeks compared with control (LETO) rats. Tadalafil treatment reduced the total urine volume from x4.4 to x1.7 and the voiding frequency from x3.9 to x1.8 of control values.

Morphological and functional changes in the bladder of OLETF rats could be a consequence of polyuria rather than the cause of increased voiding frequency. After all, the manuscript including the title should be largely rewritten.

The results of cystometry lack statistical analysis, e.g., bladder capacity, number of non-voiding contractions or peak voiding pressure. The number of experiments (animals) should be provided along with p values throughout the text.

Cystometric traces lack scale bars. In Fig1B, L-36 and O-36 traces appeared to be shown in different time scales (see the difference in the duration of voiding contractions). Please provide clearer images.

The authors did not answer why atropine completely diminished KCL-induced contractions of DSM strips. This should not be happened if experiments were carried out properly (see my comment to the original submission).

Run out of animals is not a good excuse as both OLETF and LETO rats seem to be commercially available (<http://www.hoshino-lab-animals.co.jp/English/products/OLETF_ENG..html>).

Yet, additional experiments are desirable but not mandatory in the present study.

Please check the accuracy of references (for example No.39).
